# Role of Microbial Communities and Their Functional Gene in Anammox Process for Biodegradation of Bisphenol A and S in Pharmaceutical Wastewater

**DOI:** 10.3390/toxics13040252

**Published:** 2025-03-28

**Authors:** Ruili Yang, Yonghao Sha, Zhuqiu Sun, Bairen Yang, Farheen Solangi

**Affiliations:** 1 Yancheng Institute of Technology, Yancheng 224051, China; rlyang1989@163.com (R.Y.); 18861987736@163.com (Y.S.); sunzq@ycit.edu.cn (Z.S.); 2 Research Centre of Fluid Machinery Engineering and Technology, Jiangsu University, Zhenjiang 212013, China; dr.farheensolangi@gmail.com

**Keywords:** pharmaceutical contamination, wastewater, advanced oxidation technologies, anaerobic, biphenyl A and S

## Abstract

Substantial amounts of nitrogenous (N) compounds, as well as bisphenol A (BPA) and bisphenol S (BPS), contribute to the impurities of pharmaceutical contamination (PC) in wastewater, which have detrimental effects on the environment, humans, and aquaculture. The anammox processes is primarily used to treat wastewater contamination, in which certain microbial communities play a crucial role. In this regard, the present study focuses on microbial communities and the functional genes involved in the anammox process. Further, the current study highlights the secondary (biological) and tertiary (advanced) methods; these techniques are more effective solutions for PC treatment. Anammox bacteria are the primary drivers of the wastewater’s ammonium and nitrite removal process. However, overall, 25 anammox species have been recognized between five important genera, including *Anammoxoglobus*, *Anammoximicrobium*, *Brocadia*, *Kuenenia,* and *Jettenia,* which are mainly found in activated sludge and marine environments. The group of bacteria called anammox has genes that encode enzymes such as hydrazine synthase (HZS), hydrazine dehydrogenase (HDH), nitrite oxidoreductase reductase (NIR), hydroxylamine oxidoreductase (HAO), and ammonium monooxygenase (AMO). The anammox process is responsible for developing about 30% to 70% N gases worldwide, making it a critical component of the nitrogen cycle as well. Therefore, this review paper also investigates the pathways of hydrazine, an intermediate in the anammox process, and discusses the potential way to significantly decrease the N-compound contamination from wastewater systems and the environmental effects of determined organic contaminants of BPA and BPS.

## 1. Introduction

**Figure 1:**
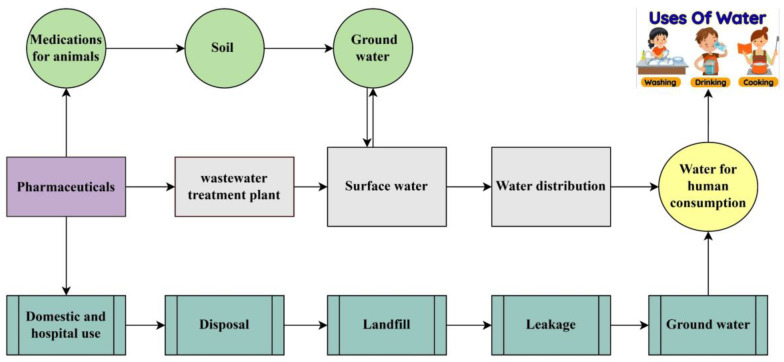
Pathways of causing pharmaceutical contaminants (PCs).

Water is the most common compound on earth, and it is essential for humans and other organisms, but the availability of clean water depends on the functioning of the world [1]. Water is a major contributor to domestic and industrial purposes; therefore, it is crucial to monitor the quality of surface and groundwater [2]. Currently, water bodies are being influenced by Emerging Contaminants (ECs), which have the capability to infiltrate ecosystems and are harmful for the environment as well as humans [3,4]. Pharmaceutical contaminants (PCs) are one of the major categories of ECs that can be used to prevent human disease. They are a biologically active compounds that arise form pharmaceutical industries used for medication or disease treatment [5,6]. Personal care products (PPCPs) are mostly used to ensure better quality of life, which includes all cosmetics products and many other useful items that are utilized in our daily lives [7]. PPCPs and pharmaceuticals, both considered important sources for increasing PC in the environment, have adverse impacts on aquatic ecosystems and human health. Some pharmaceuticals can disrupt reproductive cycles in women and men by acting as endocrine disruptors. Estrogen hormone decreases gonadotropin output, leading to an atrophic effect on adult female ovaries. Several pharmaceutical pollutants increase cancer risk due to genotoxic effects observed in aquatic organisms [8]. However, the pathways leading to PC are depicted in Figure 1. Mostly, when these products are used and then disposed of, they can enter wastewater systems and finally reach water bodies. Many PPCPs are not completely removed during wastewater treatment, but there are some methods used to reduce the high environmental risk [9]. The continuous release of PCs into water bodies can lead to long-term, chronic effects on both animals and aquatic plants, disrupting their health and ecosystems over time. Antibiotic medications like ciprofloxacin and sulfamethoxazole are frequently detected, with concentrations that contribute to expansion of antibiotic-resistant bacteria [10]. Antidepressants like fluoxetine (Prozac) were detected in aquatic environments; they can have harmful effects on biodiversity specially on fish [11]. Pharmaceutical residue concentrations in the environment vary and are mostly influenced by human activity, production waste, and wastewater method capabilities [12]. However, bisphenol A (BPA) and bisphenol S (BPS) are also major environmental concerns because of their harmful effects on human health and their estrogenic and genotoxic effects [13,14]. Both BPA (C_15_ H_12_ O_2_) and BPS (HOC_6_H_4_)_2_ SO_2_ originated from personal care products (PPCPs); they are more commonly associated with polycarbonate plastics and used as a primary raw material for the production of beverages, food cans, water bottles, and flame retardants [15,16]. Wastewater treatment confirms that PPCPs are an important source in aquatic environments because of the partial deduction of PPCPs during the treatment process. Wastewater treatment has become of greater significance due to the growing concern about xenobiotics in water resources and how to eliminate them from water [17].

Various cost-effective techniques exist to prevent the release of PPCPs into the ecosystem. Certain PPCPs pose a challenge for microorganisms to break down, as pharmaceuticals were originally designed to be toxic. Some potential bacterial species have the abilities to reduce and degrade pharmaceutical pollutants, changing their complex structure into a less toxic or nontoxic form [18]. Anammox is a biological process conducted by autotrophic bacteria, also known as “anammox bacteria”, which removes N compounds from the wastewater and converts ammonium (NH_4_^+^) into nitrite (NO_2_^−^). This process is involved in N-cycle reactions; for example, nitrite accepts electrons and reacts with ammonium to make molecular N in anaerobic conditions [19].

Anaerobic autotrophic spherical microorganisms, which are classified within the anammox bacterial group, belong to the order *Brocadiales* within the phylum *Planctomycetes* [20]. Researchers have found that over 25 anammox species have been identified across five genera, including *Anammoxoglobus*, *Anammoximicrobium*, *Brocadia*, and *Kuenenia*, *Jettenia* which are primarily found in activated sludge and various freshwater and marine environments [21]. Researchers have observed *Anammoximicrobium* genera in conditions with specific organic matter, and *Scalindua* genera are frequently detected in natural habitats, particularly in marine sediments and oxygen minimum zones [22]. In this regard, the present review focuses on microbial communities and their functional genes, as well as their role in treating wastewater contamination through anaerobic ammonium oxidation (anammox) [23,24].

Further, this study explores the effects of bisphenol A (BPA) and bisphenol S (BPS) on the environment and human health. However, there are different treatments and methods that are widely used to treat the pharmaceutical wastewater contamination. In the present study, both secondary (biological) and tertiary (advanced) methods are described to remove pharmaceutical wastewater contamination. In addition, the mechanisms of the microbes’ community, which significantly contribute to the anammox process, are also explained. Furthermore, we obtain a comprehensive understanding of the hydrazine intermediate pathways and explore strategies for mitigating their detrimental impacts on the environment, human health, and aquatic ecosystems.

## 2. Influence of Pharmaceutical Wastewater Contamination on Human Health and Ecosystem

**Figure 2:**
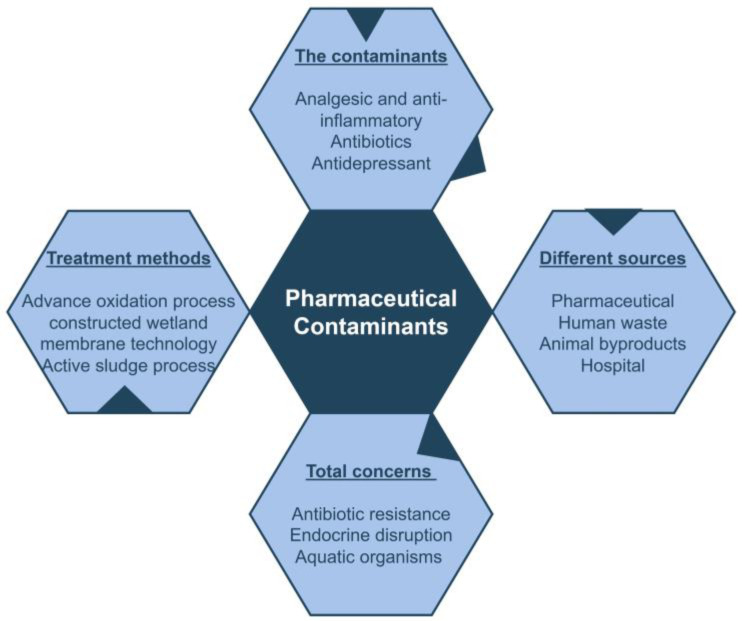
Creative general idea of pharmaceutical contaminants (PCs).

Pharmaceutical contamination of wastewater can lead to the development of certain microbiological diseases. Pesticides, medicines, industrial waste, chemical pollutants, heavy metals, and agricultural runoff are some of the factors that contribute to wastewater pollution and cause the harmful effects for both human health and the aquatic ecosystem (Figure 2) [25]. Nowadays, the excessive use of pharmaceutical products and peroxides has raised worldwide concerns due to some of these compounds entering the environment. About 100,000–200,000 tons of antibiotics and 7 million tons of peroxides are annually used in the world [26]. Pharmaceuticals encompass a wide variety of biological compounds used for treating diseases and infections. The disposal of substances, birth control hormones, painkillers, and other medicines in water bodies is extremely concerning [27]. During the pandemic, there was an increase in the use of specific drugs, which in turn lead to an increase in the number of PCs in wastewater. Over the past few decades, the production and consumption of pharmaceuticals and PPCPs have significantly increased, and therefore the concentration of these compounds in wastewater has escalated rapidly. For the entire ecosystem, a hypothetical threat occurs when the PCs are present and become absorbed into living organisms [28].

These PCs come from different areas such as households and sewers (from humans and animals), agriculture waste runoffs (from fertilizers and pesticides), hospital waste (from hospitals and dispensaries), and pharmaceutical industries (from factories and industries). They enter in our environment and threaten the entire ecosystem [29,30]. The hospital effluents, including active drugs, solvents, metabolites, sanitizers, heavy metals, and hazardous chemicals, can persist in the environment for centuries [31]. Due to their high mobility in the liquid phase, they threaten nature [5,27,32]. Therefore, it is important to ensure effective treatment before releasing these products into water bodies.

### Some Microbial Pathogen and Bacterial and Fungal Diseases Caused by Wastewater Pollution

Currently, many classes of pharmaceuticals are available, categorized on the basis of their curative applications, but they contribute to several microbial pathogen and bacterial diseases caused by pharmaceutical wastewater pollution. Pharmaceutical pollution in wastewater can alter the microbial communities. Such changes can cause harmful bacteria to grow and microbiological disorders to appear in the human body, for example, imicrobial diseases such as *Methicillin-resistant Staphylococcus aureus* (MRSA) infections, multidrug-resistant *Pseudomonas aeruginosa*, and *Klebsiella pneumoniae* infections. When antibiotics are present in wastewater, they select for bacteria strains that are resistant to them. This makes it easier for resistance genes to be passed from one cell to another using plasmids and transposons. This process enhances the survival and spread of multidrug-resistant pathogens, leading to infections in humans [33]. Gastrointestinal infectious diseases are *Clostridioides difficile*-associated diarrhea and gastrointestinal infections caused by *Escherichia coli*. Residual pharmaceuticals, including nonsteroidal anti-inflammatory drugs (NSAIDs) and antibiotics, disrupt natural microbial communities in wastewater, allowing pathogenic bacteria to outcompete beneficial microbes. This process increases the risk of gastrointestinal diseases [34]. Meanwhile, fungal infectious diseases are Candidiasis (*Candida auris*) and Aspergillosis (*Aspergillus fumigatus*). The pollution of wastewater with antifungal drugs like fluconazole leads to the selection of resistant fungal pathogens. This pollution mostly occurs in hospital wastewater, where persistent exposure to low doses of antifungal agents promotes the emergence of drug-resistant fungal strains [35]. Researchers have used a variety of biological and physical chemical treatments to remove PCs from wastewater with different time interval and improve their quality [36,37].

Pharmaceuticals are divided into different types or groups according to their chemical structures, modes of action (interacting and binding to biological targets), and therapeutic applications in the treatment of diseases. Human health issues are often linked to groundwater contamination and environmental pollution throughout the world; the idea shown in Figure 2. Improper handling and persistence of plastic trash result in the accumulation of microplastics in the environment, the transmission of pollutants, and the leaching of hazardous additives [38]. Behavior and genetic traits of living things may also change as a result of PC exposure [12]. Chloroquine, which has a calcium calmodulin-mediated response, disrupts estrous cyclicity as follicular steroidogenesis and pituitary hormone secretion also depend on the same response. Additionally, PCs in drinking water may adversely affect elderly individuals and those with liver and kidney failure. Further, PCs in drinking water is a main cause of stomach problems in newborn babies. The presence of estrogens in drinking water reduces male fertility; it may also raise the risk of testicular and breast cancer [39]. Due to the availability of PCs in water bodies, many harmful effects have been noticed in humans and animals.

Therefore, it is necessary to create a dynamic plan to have some efficient treatment methods for extraction of PCs from wastewater. In several previous studies, various techniques have been used for extraction of PCs and the treatment of pharmaceutical wastewater, but in most cases, a very important role was assigned to a wastewater treatment plant (WTP) using biological methods [40,41,42]. Therefore, in this review, a detailed discussion about biological methods appears along with advanced methods of pharmaceutical wastewater treatment in Section 4.

## 3. Effects of Bisphenol A (BPA) and Bisphenol S (BPS) on Human Health and Wastewater Quality

Endocrine disruptors are synthetic and natural compounds that can interfere with hormone function, mostly dangerous to the endocrine system [43]. Due to modern lifestyles, these harmful chemicals are widespread in the environment and include endocrine disruptors BPS and BPA. Living things are exposed to these dangerous substances either directly or indirectly. In wastewater, pharmaceutical compounds are frequently detected, with different concentrations based on wastewater treatment efficiency, regional pharmaceutical usage, and environmental conditions. Previous research studies identified several classes of pharmaceuticals present in wastewater effluents. The Non-Steroidal Anti-inflammatory Drugs (NSAIDs) used for pain relievers like ibuprofen and diclofenac have been found in wastewater, the concentrations ranging from nanograms to micrograms per liter [44]. Aleksandr Dianin first identified biphenyl A as a hazardous compound in 1891; after that, Zincke synthesized it through acetone concentration with two phenol derivatives in 1905. In 1940, the mid-20th century saw a rapid increasing trend of polymers, such as polysulfone, epoxy resins, and polycarbonates, along with BPA. These polymers served as both a barrier to prevent polymerization and an antioxidant. The polymers also facilitated the synthesis of flame-retardant polymers like tetrabromo bisphenol-A [45]. In 2015, the demand for polycarbonates was 34%, while the demand for epoxy resins was 64%; after that, the anticipated demand is expected to increase with each subsequent year [46]. Recently, BPA and BPS have been the important concerns of researchers and the general public due to their ubiquitous nature [47]. Further, bisphenol A (BPA) finds its way into food packaging, receipts, and plastics, posing potential health risks due to its endocrine-disrupting properties [48]. BPA is harmful as an endocrine-disrupting chemical (EDC) which causes hormonal changes as well as changes in enzyme synthesis, release, and transport. The BPA inhibits the endocrine system’s activity by competing with endogenous hormones for binding to transporter proteins. This chemical also affects neuroendocrine function, leading to physiological disruptions in various organs. An earlier study showed that BPA reduces testosterone levels in males and increases estrogen serum levels in females [49]. A previous study indicated that BPA acts on the mammary gland through ER beginning in the fetal mesenchyme, altering gene expression and the development of adipose and other cells of the stromal section and promoting the growth of the fetal mammary epithelium [50].

Another study showed that BPA can prompt carcinoma of the mammary gland in sensitive rat strains, providing evidence that it is a complete carcinogen. Another example is the female reproductive system, where BPA has negative effects on the ovary and uterine epithelium [51]. In addition, adult exposure to BPA significantly lowers spermatogenesis, which is similar to the effects of oestradiol and oestrogenic drugs. In order to prevent androgen-stimulated prostate tumor growth, hydroxyflutamide is used as an antiandrogen. However, a research study described that BPA lowers the quality of oocytes, blocks steroidogenesis, and changes how oocytes mature and undergo meiosis [52]. BPA also disrupts male germ cells, causing similar effects on oocytes during early development and again after maturation. In other words, numerous studies demonstrate that BPA has an impact on metabolic disorders [53], including effects on the pancreas, adipose tissue, and liver, as well as glucose intolerance [54].

### 3.1. Mechanisms of BPA and BPS Removal in Anammox Systems

Removal of BPA and BPS in the anaerobic anammox system involves several complex mechanisms. In this part, we discuss the primary processes that contribute to BPA and BPS removal in anammox reactors.

#### 3.1.1. Adsorption Mechanisms

Initially, the BPA and BPS molecules are adsorbed into biomass and sludge particles within the anammox reactor, allowing for gradual biodegrading but reducing their immediate bioavailability [55]. In this process, extracellular polymeric substances (EPSs) and contaminants play an important role. A research study reported that BPA was linked with tryptophan-like proteins in EPS through enthalpy-driven reactions [56]. Furthermore, EPS composition and structure can be expanded directly to electron transfer capacity, which is considered favorable for subsequent biodegradation processes [57].

#### 3.1.2. Biodegradation Processes

In this process, anammox bacteria and co-existing microbial species may enzymatically break down BPS and BPA through reductive pathways and oxidation [58]. However, these types of study which are involved in biodegradation processes for BPA and BPS are limited in anammox systems, which indicates the need for further research in this area.

#### 3.1.3. Transformation Products

Through the treatment process, several transformation products such as hydroquinone and phenolic derivatives have been found [55]. These intermediates may be further degraded to simpler and less toxic compounds. A research study states that within the treatment system, BPS is not only adsorbed but can also be structurally transformed [59].

#### 3.1.4. Electron Transfer Mechanisms

A study highlighted that the transfer of an electron mechanism is important in the removal of BPA and BPS [60]. A previous study showed that direct interspecies electron transfer (DIET) in between anammox bacteria and other microorganisms in the reactor can facilitate the degradation of these contaminants [61]. This process is used on conductive materials or naturally occurring electron shuttles to increase the transmission of electrons in between species, eventually to enhance the whole removal efficiency of BPS and BPA. This study was very useful and filled an important gap in the topic of transfer of the electron mechanism in the removal of BPA and BPS.

#### 3.1.5. Synergistic Effects

The availability of other pollutants in pharmaceutical wastewater can have synergistic effects in the removal of BPA and BPS in anammox systems. Another research study observed that co-existence of particular antibiotics might increase the degradation of BPA by altering the microbial community structure and stimulating the expression of relevant enzymes [62]. The delicate balance between anaerobic ammonium oxidation (anammox) and dissimilatory nitrate reduction to ammonium (DNRA) is demonstrated by a recent study on their synergistic interactions. The study shows how the niche divergence, organic carbon concentrations, and influent loading rates of anammox species affect these interactions [63]. Understanding removal mechanisms is vital for evaluating the efficiency and safety of anammox-based treatment processes for pharmaceutical wastewater containing BPA and BPS.

This shift has led to exploring other chemical solutions. Due to their potential health risks and endocrine-disrupting properties, companies are gradually removing their use of BPA and BPS. BPA has been associated with adverse effects such as hormonal imbalances, reproductive system issues, and increased cancer risk [48]. We already discussed BPA above; here, we discuss BPS. BPS is used in various industrial applications, including electroplating solvents, cleaning agents, and as a key component in phenolic compounds [60]. Furthermore, BPS is also used for development of thermal paper; BPA-free paper could be used [64]. BPS has been detected in many products that are used in our daily life (toothpaste, hair care products, lotions, body wash, makeup) [65], paper products (tickets, envelopes, currency, airplane boarding passes) [64], and food (preserved foods, dairy products, meat products, vegetables) [65]. In indoor dust samples, BPS was detected at a concentration of 0.34 μg/g, while BPA was found at 1.33 μg/gs [66]. Moreover, it was noticed that in many markets, unapproved chemicals are placed with original chemicals; for this matter, such chemical analogs should be assessed before they arrive or are replaced for toxic chemicals.

It has been reported that these chemicals are more dangerous for humans compared to the original ones [67], flame-resistant chemicals [68] and pesticides [69]. Asia Pacific holds the largest BPA manufacturing market, accounting for about 52% of the market share, with the USA and Western Europe accounting for 36%. The production, use, and disposal of BPA in the environment are the primary factors contributing to its inclusion in ecological systems. It is important to find alternative solutions due to BPA’s harmful effects on health. Researchers have conducted numerous studies in recent years to identify a better alternative to BPA that shares the same properties [70]. Similarly, BPS, introduced as a BPA substitute, has demonstrated comparable endocrine disruption in numerous studies, raising concerns about its safety. As a result, currently, manufacturers are exploring alternative bisphenol analogs (e.g., bisphenol F and bisphenol AF), although preliminary research indicates that these substitutes may also exhibit similar detrimental effects on human health and the environment [71]. Additional research on this group of compounds is also recommended, to evaluate their potential impact on human health. The authors suggest that alternative-based (BPA-free) products could serve as viable substitutes for BPA-containing materials. Stricter regulations on BPA due to its environmental and health risks have led to a growing demand for safer alternatives in research and industry. In response, researchers have developed several “bisphenol analogs” as potential substitutes for BPA. However, further studies are needed to evaluate their safety and effectiveness, as these compounds may serve as viable alternatives [72].

## 4. Approaches for Clean Pharmaceutical Wastewater

### 4.1. Biological Treatment

**Figure 3:**
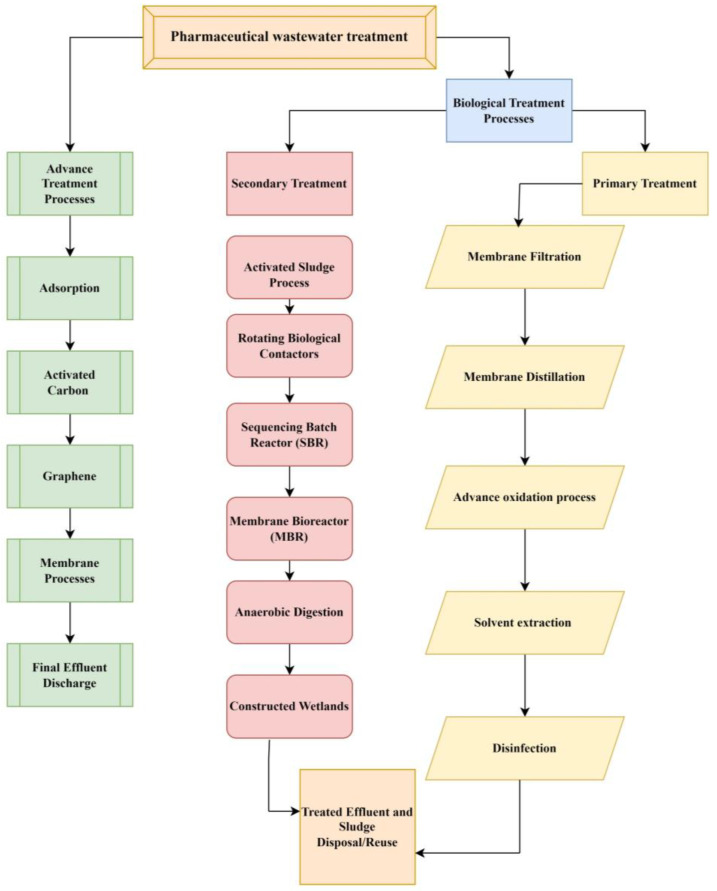
Different treatments for PC including biological and advanced methods.

There are various ways and strategies present to treat pharmaceutical wastewater contamination including physical, chemical, and biological methods. The wastewater purification process consists of five main steps such as preliminary treatment (physical and mechanical processes), primary treatment (chemical and physical–chemical processes), secondary treatment (biological and chemical processes), and tertiary or advanced treatment (physical and chemical processes). However, the current study discusses biological and advanced techniques to treat the contamination of wastewater (Figure 3) [73]. Decomposition of ECs into tiny molecules or even to water and CO_2_ using biomineralization employing microorganisms like algae, fungi, and bacteria leads, through microbial metabolism, to the degradation of nutrients like nitrogen and phosphorus as well as organic matter [74]. Biological treatment includes solvent extraction, improved oxidation procedures, membrane distillation, biological membrane filtration, and disinfection. Further, biological treatment methods are commonly divided into aerobic processes, anaerobic processes, and combinations of anaerobic–aerobic processes [75]. Several advanced anaerobic strategies, including the anaerobic membrane bioreactor (AnMBR), the UASB, the anaerobic sequencing batch reactor (AnSBR), the moving bed biofilm reactor (MBBR), and other hybrid technologies, have demonstrated their effectiveness in pharmaceutical treatment [76]. Because wastewaters have a high organic content, the anaerobic method—which requires fewer nutrients and little space—seems to be an efficient, economical, and effective way to break down medications [77]. The membrane bioreactors and anaerobic treatment plant batch reactors are the key technology of treatment plants [78]. Research is being conducted on biological treatment for plants, which creates renewable energy in methane, and it is an environmentally friendly treatment. Furthermore, the feasibility of using anaerobic digestion and sulfamethoxazole pharmaceutical wastewater treatment is limited; research has explored the possibility, but it these types of treatments remain largely uninvestigated [79,80].

The recovery of carbon in renewable biogas is important in anaerobic digestion and is increasing; it can lead to higher methane yields [81,82]. A study demonstrated the low degradation efficiency, low mineralization efficiency, and the application of anaerobic techniques in antibiotics [83]. Other studies reported that applying energy to stimulate anaerobic systems can significantly enhance the mineralization of chlorine-substituted pollutants and persistent nitrates while simultaneously improving energy recovery [55,84]. Research found the elimination of antibiotic contamination risk about chloramphenicol by 54% to 90% could be possible by increasing the voltage and more than tripling methane production [85]. Additionally, another study also reported that electrical stimulation of dominant functioning bacteria further boosts methane generation and increases antibiotic resistance [86]. However, advanced research is still not capable of evaluating long-term whether anaerobic sludge treatment is cost-effective for pharmaceutical wastewater treatment [87]. The Up-flow Anaerobic Sludge Blanket (UASB) reactor has proven to be an effective and cost-effective method for treating some hazardous substances and urban wastewater [88]. Thus, antibiotics like carbamazepine (CBZ) have displayed significant resistance to anaerobic treatment. An earlier study found a chemical oxygen demand deletion of 46% in anaerobic conditions [89]. Another study discovered contaminations of the Up-flow Anaerobic Sludge Blanket (UASB) reactor. However, the anaerobic process often has a slow growth rate compared to aerobic complements. Long start-up periods and anaerobic microorganism rates are commonly associated with pharmaceutical wastewater treatment [90]. Previous research investigated the UASB treatment combined with wetlands constructed to treat the discharge of pharmaceutical wastewater [91]. Around 95% removal of pollutant was achieved with these methods combined, UASB treatment and the method of constructed wetlands [92]. In addition, research explored granular activated carbon (GAC) and zero-valent iron (ZVI) to enhance anaerobic digestion of pharmaceutical wastewater [93]. The study result reveals that combined application of GAC and ZVI could be improved by around 13% of COD and 11% of methane gas production [84]. Integrating anaerobic wastewater treatment techniques improves environmental sustainability and supports eco-friendly pathways for biofuel production [94]. These studies succeed in providing scientific results, but research is limited and solutions remain largely unknown. These types of treatments need further studies, which will help to better understand and respond to various environments. Keeping this in view, the present study also focuses on advanced techniques for cleaning wastewater contamination [95].

### 4.2. Advanced Treatment

#### 4.2.1. Adsorption Activated Carbon (AC)

The adsorption process is one of the most efficient, easiest, and most economical methods for removing pharmaceutical contamination [96]. This advanced method is highly efficient, has low operating costs, and easily adapts to the environment. Adsorption performance is directly dependent on the quality and cost-effectiveness of the adsorbent, including graphene oxide, zeolite, and activated carbon [97]. The adsorption process is widely used for the efficient removal of trace organic pollutants from water due to its simplicity, high effectiveness at low concentrations, and minimal waste generation [98]. Many adsorbents are available; wastewater removal of PPCPs by activated carbon (AC) is attractive and used not only in laboratory studies but also in full treatment plants and pilot plant studies [99,100]. A previous study summarizes several studies on efficient removal of PPCPs with AC [9]. In addition, many mechanisms are involved in the removal of PPCPs by the AC method, described in [101]. The current study follows previous studies in a style of review-based summary and mentions the overall removal efficiency percentages of PPCPs from wastewater using nanofiltration (NF) and NF with adsorption pre-treatment methods, including Purolite and Granular Activated Carbon (GAC). Table 1 provides a summary of the removal efficiencies of 10 PPCPs, comparing the performance of NF alone versus NF combined with adsorption pre-treatment.

#### 4.2.2. Graphene

Graphene consists of a single-layer, two-dimensional arrangement of carbon atoms in a hexagonal pattern resembling a honeycomb structure [104]. It is obtained from graphene oxide that is produced through the oxidation of graphite. Both graphene and graphene oxide have larger specific surface areas than AC, making them potentially better at adsorbing pollutants [105,106]. Graphene oxide could be utilized alone and in combination with other materials, such as activated carbon and nanoparticles, to enhance its performance in clean water contamination treatment [107]. Various forms of large surface area graphite are efficient for removing the different forms of wastewater contamination. Trimethoprim and metronidazole are some examples of pharmaceuticals that remove contamination from wastewater through an adsorption process using graphite. The adsorption abilities of graphite are lower than those of graphene oxide [108]. Generally, 218 mg/g of the trimethoprim is adsorbed by graphene oxide under room temperature at pH 10, and other types of graphite are not able to adsorb pharmaceuticals up to that capacity [109]. The nano-sheets of these materials tend to clump together in water due to strong interactions between the layers and their adsorption efficiency, which reduces their adsorption range of 278 mg/g for the removal of diclofenac from water. Because it is not harmful to living things, this substance is also an excellent adsorbent in water. One way to address this issue is by attaching graphene and graphene oxide nanosheets to low-cost substrates [110]. These materials have been tested in lab studies to absorb pharmaceuticals and personal care products, mostly in batch experiments using synthetic wastewater with much higher PPCP concentrations than found in real wastewater [103]. As a result, the findings may not accurately reflect real-world conditions. For more reliable results, PPCP adsorption by graphene and graphene oxide should be evaluated in pilot-scale and full-scale plants using real wastewater conditions. This will provide a more accurate assessment of their performance in practical, large-scale applications.

#### 4.2.3. Membrane Process

The membrane processes to remove pollutants from water through water treatment plants (WTPs) use different processes: ultrafiltration process (UF), reverse osmosis process (RO), and nanofiltration process (NF). In these processes, different sizes of pores are used, ranging from 0.1, 0.01, and 0.001 µm, respectively [111]. Large pores consume less energy and operating costs, but they also remove a small number of pollutants. The membrane removal of PPCPs occurs through mechanisms such as processes of absorption, exclusion, and electrostatic repulsion [112,113]. The removal of PPCPs depends on various factors such as charge, size, and hydrophobicity. For suspended solids, MF and UF membranes typically have large pore sizes. While microfiltration (MF) and ultrafiltration (UF) membranes cannot remove PPCPs due to their molecular weights (MWs), which typically range from 200 to 800 Da (approximately 0.000025 to 0.0001 μm), the molecular weight cut-off (MWCO) of MF and UF membranes falls in the several thousand Dalton range [112]. However, PPCPs can be effectively removed by nanofiltration (NF) and reverse osmosis (RO) methods, as determined in [114]. Commercially available RO and NF membranes differ in properties such as charge, MWCO, and hydrophobicity/hydrophilicity, which impact their efficiency in removing specific PPCPs. Research studies have reported that PPCP removal performance depends on different types RO and NF membranes [112]. Another study has examined PPCP removal using synthetic and ultra-pure water [115].

A research study conducted in northern Spain checked the combined effects of the UF and RO pilot plant. In this study, secondary treated effluent and raw municipal wastewater were used [115]. In total, 12 PPCPs (atenolol, bezafibrate, caffeine, fenofibric acid, gemfibrozil, hydrochlorothiazide, ibuprofen, nicotine, ofloxacin, naproxen, furosemide, and N-acetyl-4-amino-antipyrine (4-AAA)) were observed. In this study, it was observed that removal of PPCPs was very low around >20 when using only UF, but when UF was followed by RO, about a 99% removal was succeeded. The system was operated at 11 bars because of low TMP. A research study conducted on the removal of PPCPs using nanofiltration (NF), utilizing microfiltration (MF)-treated water from a water treatment plant (WTP) in Sydney, which processed domestic sewage and storm water [116]. The study monitored 10 types of PPCPs with molecular weights ranging from 119 to 296 g/mol and either negative or neutral charges. An NF90 membrane was employed, characterized by a molecular weight cut-off (MWCO) of 90–200 Da, moderate hydrophobicity, and a negative charge. This study results of laboratory analysis show that the removal rate was between 35% and >98%. Seven of the ten monitored PPCPs showed a removal rate of over 90% using NF alone. This high removal efficiency is due to the negative (N^−^) charge of the NF membrane, which filters for four N^−^ charged PPCPs (gemfibrozil, diclofenac, naproxen, and ibuprofen), regardless of their molecular weights. During the NF process, PPCPs could be removed by adsorption onto organic material. Both saccharin and benzotriazole have high rates of rejection, 88% and 35%, respectively; therefore, they were not removed. These PPCPs pass through larger membrane pores because both have low rates of removal and neutral charges of 183 and 119 g/mol, correspondingly. The improper disposal of this concentrate poses significant health risks to non-target species, especially in aquatic ecosystems, potentially exceeding those of the original wastewater. Therefore, it is crucial to treat this concentrate thoroughly before its discharge into water bodies.

### 4.3. Advanced Oxidation Processes (AOPs) with Biological Treatment Methods

However, advanced oxidation processes (AOPs) remain one of the most promising, efficient, and environmentally friendly methods for eliminating persistent organic pollutants (POPs) due to their chemical stability [117]. Their effectiveness has been well documented in wastewater treatment across industries such as textiles and pharmaceuticals, as well as within circular economy models worldwide. Advanced oxidation processes based on sulfate radicals (SR-AOPs) have developed as an effective method for eliminating organic contaminants by generating highly reactive oxidizing species, primarily sulfate radicals (SO₄•) [118]. These radicals can oxidize and stabilize a wide range of organic pollutants by facilitating electron transfer through mechanisms such as addition, substitution, oxidation, and bond cleavage. These radicals can oxidize and stabilize a broad variety of organic contaminants [119]. More pollutants can be broken down by sulfate radicals than by hydroxyl radicals (HO•), which have a redox potential of (1.8–2.7 V). This is because sulfate radicals have a higher redox potential (2.5–3.1 V), a wider pH range, and fewer unwanted byproducts [120]. The main mechanism for oxidizing organic pollutants involves reactions with hydroxyl radicals. The two main steps that all AOPs usually take are making strong oxidants and then reacting those oxidants with organic pollutants in water [121]. Wet air oxidation (WAO) is one of the most important techniques used for wastewater treatment, breaking down refractory organic pollutants. Zimmermann came up with wet air oxidation (WAO), which is one of the most technologically and economically feasible AOPs for wastewater treatment. Each AOP’s suitability changes according to the effluent’s organic content and flow rate. Some AOPs, like ozonation and wet peroxide oxidation (WPO), work better when there is low flow and low organic loading. On the other hand, biological treatments work better when there is high flow and low organic loading [122]. WAO can be used to treat wastewater with a lot of organic matter that is flowing quickly because, unlike other AOPs, it can be used for both biological and incineration processes. WAO can handle wastewater with an initial chemical oxygen demand (COD) between 20 and 200 g/L, which makes it appropriate for both diluted and concentrated wastewater streams [123]. Several additional treatment processes have been utilized for the removal of organic contaminants, including filtration, ion exchange, magnetic separation, coagulation–flocculation, membrane processes, biodegradation, chemical oxidation, adsorption, and nanofiltration [73]. A light-driven AOP method was employed to treat ibuprofen, ciprofloxacin, and carbamazepine in wastewater, achieving removal efficiencies of 89.83%, 100%, and 80.4%, respectively, within 40 min. These findings highlight the significant potential of AOPs in addressing pharmaceutical wastewater contamination [124].

## 5. Mechanisms of Microbial Communities Involved in Anammox Process

Extensive usage of antibiotics could lead to the presence of different antibiotics in pharmaceutical wastewater and aquaculture [24]. Excessive nitrogen compounds released from the source of pharmaceutical and industrial waste into the aquatic environment can cause eutrophication. The dilemma of depletion of energy in wastewater treatment has been resolved by the discovery of the anaerobic ammonium oxidation (anammox) process [125]. Developing an effective process for N removal can improve the situation with environmental losses and also be helpful for aquatic systems [126]. If ammonia is discharged improperly in wastewater, it can have detrimental effects on aquatic ecosystems. It is toxic to living organisms and contributes to eutrophication in water bodies, leading to oxygen depletion and harmful algal blooms [127].

**Figure 4:**
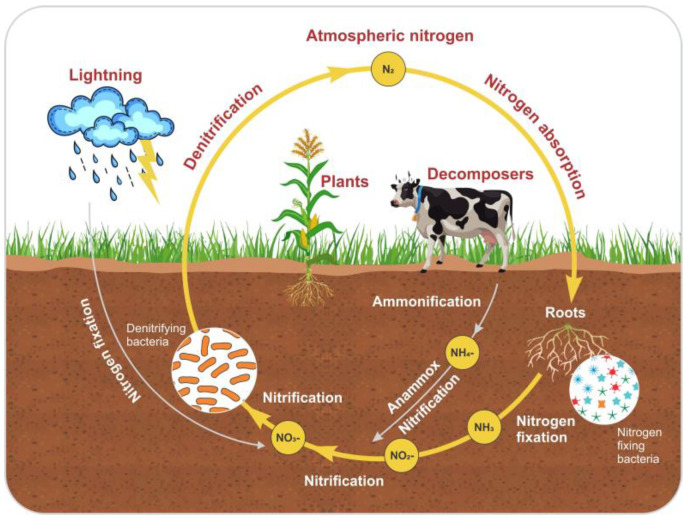
The nitrogen cycle and anammox process.

Anammox is considered a unique process for N removal. This process simultaneously removes nitrite and other types of wastewater pollutants. Figure 4 shows the N cycle and the anammox process. Furthermore, the effects of antibiotics on the anammox system have been extensively studied in recent years [128]. Several decades ago, researchers made significant efforts to explore the mechanisms involved in and responsible for anammox processes that are facilitated by microbial communities and provide the most energy-efficient process for N removal from wastewater [129]. They were compared to the traditional method, which is used for nitrification and denitrification and involves aeration treatments with the addition of exogenous organic carbon sources, which lead to high costs for wastewater treatment [130]. However, this process was initially discovered in a denitrifying fluidized bed reactor [131]. In the chemoautotrophic biological process, the NH_4_^+^ is converted into elemental N_2_ gas with NO_2_^−^ as the electron acceptor [132]. Nitrification is a basic reaction that contains two main steps: the oxidation of ammonia (NH_4_^+^) to nitrate (NO_3_^−^) via nitrite (NO_2_).

The sensitivity of anammox bacteria (AnAOB) to environmental contaminants (e.g., antibiotics, salinity, and heavy metals) limits the practical application of anammox technology [133]. However, different kinds of reactions occur when various types of bacteria are present. Firstly, the key enzyme ammonia monooxygenase (AMO) catalyzes two important types of microbial groups: ammonia-oxidizing bacteria (AOB) and ammonia-oxidizing archaea (AOA). Other microbial groups include nitrite-oxidizing bacteria (NOB), complete ammonia oxidizers (comammox), and denitrifying ordinary heterotrophic organisms (DOHOs), all of which contribute to nitrite and denitrification in the nitrogen cycle.

Additionally, both polyphosphate-accumulating organisms (PAOs) and glycogen-accumulating organisms (GAOs) improve biological phosphorus removal [134]. The relationship between AOB and wastewater contamination changes is difficult to understand. There are several combined impacts existing, including physiological microorganism processes and environmental factors. The examination of bio conservation mechanisms through AOB involves molecular, cellular, and community levels. Further, the maximum dissolved oxygen demand COD concentrations severely inhibit the activity of ammonia-oxidizing bacteria (AOB) and anammox bacteria by creating unfavorable conditions for their growth and metabolism. At the same time, these conditions promote the rapid proliferation of heterotrophic bacteria, which compete for nutrients and further disrupt the nitrogen removal process [135].

It has been found that the activities of AOB and anammox bacteria decline when carbon and nitrogen C/N ratios are recorded at 2.7 and 2.9, whereas a lower C/N ratio of 2.6 enhances nitrate-to-nitrite transformation efficiency, achieving up to 42%. A previous study confirmed a total nitrogen (TN) removal rate of 0.28 kg N/(m^3^·d) and a nitrate removal efficiency ranging from 30% to 60% [136]. In the study, nitrate was produced as an intermediate by the anammox reaction and then reduced back to nitrite by denitrifying anaerobic methane oxidation (DAMO) archaea. This combined activity of anammox bacteria and DAMO archaea contributed to nitrite removal, with anammox accounting for about 90% and DAMO archaea contributing around 10%. Greater percentages of wastewater contamination treated by anammox processes are presented in Table 2.

According to AMO enzymes, structural and functional characteristics inhibit the influence of contamination by primary metabolic reactions, which are involved at the molecular level. Further revising contamination bio conservation reactions and transformation products (TPs) formed by clean cultures of aerobic ammonia-oxidizing bacteria allows us to evaluate the molecular and cellular reliability of contamination of biotransformation by AOB at the cellular level [140]. Abundances and presence of AOB involve different reactions. At the community level, conservation of contamination by AOB NH_4_^+^ elimination rates (rNH_4_^+^) and active parameters (sludge retention time (SRT), hydraulic retention time (HRT), and dissolved oxygen demand (COD)) in systems is commonly helpful to investigate the connection between AOB and wastewater contamination. At the end of bio conservation, at three different levels (molecular, cellular, and community), there are potential approaches to increase the removal of contamination during wastewater treatment. Overall, it is determined that AOB can play a role in converting NH4^+^ into the elemental form in the wastewater contamination conversion treatment process [141].

## 6. Relationship Between Anammox Bacteria and Wastewater Contaminations

Numerous natural environments, including inland lakes, agricultural soil, and coastal deposits, exhibit abundant anammox bacteria. The anammox process is responsible for about 30% to 70% of global gaseous nitrogen production, making it a critical component of the N cycle. In addition, these ecosystems account for 50% of N loss [142]. A recent study showed the interaction between anammox and denitrification in aquatic ecosystems. These study findings suggest that anammox contributes significantly to nitrogen loss with its relative importance, while the amount of nitrogen loss varies based on environmental conditions [143]. Anammox’s contribution to N gas production varies by environment because of a number of factors, including the amount of organic carbon present, the availability of oxygen, and the composition of the microbial population. Anammox is predominant in some marine sediments and oxygen-minimum zones, while denitrification may be more prevalent in other environments. This variation highlights how crucial context-specific research is to precisely estimating the contributions of various processes.

**Figure 5:**
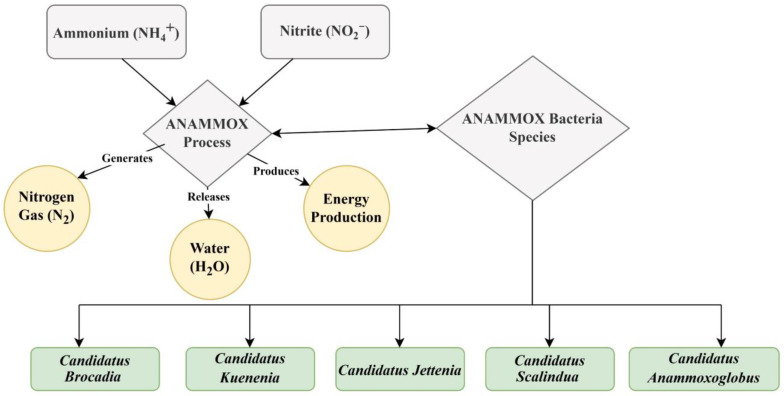
The diagram shows the anammox process and their bacterial group.

Anammox bacteria have been discovered in six genera, such as *Anammoxoglobus*, Anammoximicrobium *brocadia Jettenia*, and *Kuenenia*, which originated in activated sludge, and *Scalindua,* commonly found in marine environments in oxygen zones [144]. *Candidatus*’ holds a unique position because it does not exist in pure culture and requires cultivation in an artificial environment, specifically a laboratory. Understanding the role of these bacteria and their function is critical for the key biological mechanisms of anammox reactions shown in Figure 5. The reactions with two intermediates (hydrazine and NO) and three key enzymes, such as hydrazine synthase (HZS), hydrazine dehydrogenase (HDH), and nitrite oxidoreductase reductase (NIR), were found to be responsible for the anammox metabolism [145].

*Brocadiales* plays an important role in various genera in the anammox process, which is a sustainable way to remove N from wastewater. These bacteria, especially *Candidatus brocadia* and *Candidatus kuenenia*, are used as chemical energy-demanding treatments for wastewater. In addition, when converting ammonium to nitrogen gas, these bacteria help to maintain the balance of nitrogen. Their use in bioreactors has transformed nitrogen exclusion, leading to more efficient and eco-friendly wastewater management systems [73]. *Candidatus brocadia,* discovered as the first anammox bacteria, helps to reduce nitrogen pollution and decrease the need for carbon and oxygen during the wastewater treatment process [146]. The use of bioreactors has transformed nitrogen exclusion and created more efficient and eco-friendly wastewater management systems. *Candidatus brocadia,* discovered as the first anammox bacteria, helps to reduce nitrogen pollution and decrease the need for carbon and oxygen during the wastewater treatment process. While *Candidatus kuenenia* is also one of the initial anammox bacteria labeled, it works as an anammox reactor. Next, *Candidatus scalindua* plays a crucial role in removing nitrogen from the marine environment and aiding in the regulation of nitrogen levels in global marine ecosystems. *Candidatus scalindua* plays a critical role and prevents excessive nutrients that could lead to eutrophication [147]. *Candidatus jettenia* is another genus of anammox bacteria, less commonly studied but still significant in nitrogen cycling. It contributes to the nitrogen removal in both natural and engineered systems, adding diversity to anammox bacterial communities. Furthermore, *Candidatus anammoxoglobus*, a less known anammox bacteria genus, has been detected in some anammox bacteria environments where other anammox bacteria are found. It also contributes to the removal of nitrogen, but compared to other anammox genera, relatively few studies have been conducted on this topic [34,137]. According to a recent study that showed the comparative abundance of *Planctomycetes*, *kuenenia* was determined to be the most dominant gene in anammox bacteria, with relative abundances showing a decreasing trend under the humic acid treatment [148]. Anammox sludge applied to the anammox reactor helped clean up pharmaceutical wastewater because the granules are thought to be resistant to harmful chemicals [149].

Therefore, an earlier study evaluated aquaculture systems and found a positive interaction between organic matter and *Kuenenia* bacteria. The metagenomes of “*Kuenenia*” and “*Brocadia*” demonstrate the high degree of similarity, which explains their simultaneous detection in numerous ecosystems [146]. Therefore, research has proven that *Brocadia* is the dominant genera in anammox process. Its more versatile metabolism and improved adaptability, compared to other bacterial groups involved in the anammox process, suggest that *Brocadia* has successfully adapted to environments rich in resources. Mostly, in the anammox process, the presence of *Nitrosomonas bacteria is* involved in nitritation, the main nitrogen removal pathway, despite a reduction in nitritation efficiency [150]. Both *Brocadia* and *Kuenenia* were primarily found in soils and freshwater ecosystems in non-salty environments. Similarly, a previous study showed that following leachate treatment, the relative abundance of the denitrification groups was low, primarily a result of the salt stress that can lower the population of these bacteria [151]. The landfills found in the genus of Alicycliphilus can employ nitrate, chlorate, and oxygen as terminal electron acceptors during the denitrification process. They can biodegrade xenobiotics under a range of oxygen circumstances thanks to their flexible metabolism [55]. Anammox bacteria exhibit reduced oxygen staining in air saturation, leading to a significant reduction in biological reaction in high-dissolve oxygen environments [152]. Previously, a research has summarized the research on the functional genes and structure of the anammox community [137]. A recent research study examined the impact of various environmental factors on metabolic pathways, as well as the effects and mechanisms of conventional environmental factors [153], the key drivers that provide information regarding microbial communities, collaboration in wastewater treatment for intermediates, and the removal of toxic substances. Table 3 summarizes the data on discovered anammox bacteria with scientific references.

### Enzymatic Pathways for Anammox

Generally, the anammox process involves five major bacteria and their important functional enzymes, with some enzymes being responsible for all reactions beneficial to N transformation. The metabolic pathways of the anammox functional enzyme are shown in Table 4.

This study highlights the roles of several enzymes, including hydrazine synthase (HZS), hydrazine dehydrogenase (HDH), nitrite oxidoreductase reductase (NIR), hydroxylamine oxidoreductase (HAO), and ammonium monooxygenase (AMO) [158]. However, an earlier study discovered that all anammox bacteria have Hydrazine Oxidase (HAO) enzymes that help change hydroxylamine into NO [145]. Anammox functional enzymes perform a specific role in NH_4_^+^ to NO_2_^−^ removal by a metabolic pathway (Table 3). Various anammox species use nitrite, nitric oxide, and ammonium in various processes, terminating in the release of N_2_ into the atmosphere. Enzyme performing at the final step of the anammox pathway is HDH, which represents the key enzyme of the process and is present in all anammox-performing bacteria [138,145,159].

**Figure 6:**
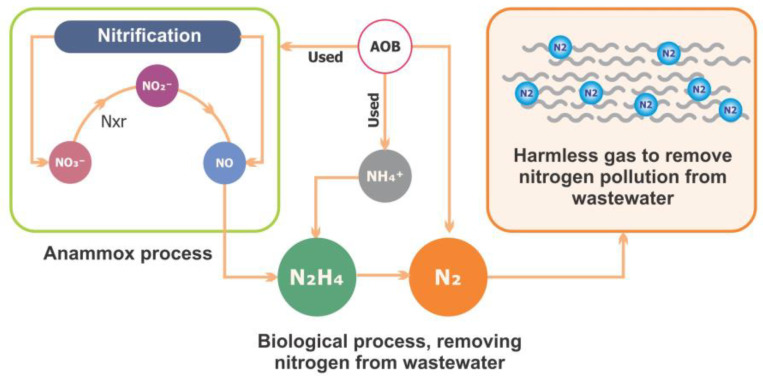
Challenges and limitations of the current nitritation-based anammox process.

The anammox species *Scalindua stuttgartiensis* and *Kuenenia* profoundly encode NIR while *Jettenia* encode NIR, whereas *Brocadia* spp. do not encode any known nitrite reductase [132]. Different genera exhibit variability in their nitrite-reducing enzyme, suggesting that they may have acquired the ability to reduce nitrite to NO at a later stage. For a long time, researchers have believed that the production of nitrate indicates the growth of anammox bacteria, and that the oxidation of nitrite to nitrate is necessary for cell carbon fixation. However, this new study shows that this is not the case; a limitation of the anammox process is also shown in Figure 6. The HZS gene codes for the hydrazine synthase enzyme. This enzyme helps make hydrazine (N_2_H_4_) from nitric oxide (NO) and ammonium (NH_4_^+^), which is an important step in the anammox reaction. Only the anammox path has the extremely reactive and energetic intermediate hydrazine. Hydrazine synthesis is important because it reduces nitrogen and eventually turns it into nitrogen gas. Anammox bacteria in environmental samples are frequently identified and quantified using hzs genes as a biomarker. The last stage in the anammox pathway, which converts hydrazine to nitrogen gas, is crucial for the release of nitrogen into the atmosphere. This could potentially increase anammox activity at 1 mg/L. Therefore, the primary determinants of nitrogen removal are the relative abundance of functional genes involved in anammox metabolism. Nitrite reductase enzymes, such as nirS and nirK, are not directly involved in the anammox pathway, but they are very important for making nitrite (NO_3_^−^), which is one of the main building blocks for the anammox process. Enzymes that convert nitrate (NO_3_^−^) to nitrite (NO_2_^−^) are encoded by these genes. Nitrite is the substrate that anammox bacteria use. In situations where nitrite is scarce, additional microbial communities with nirS and nirK genes can assist in preserving the nitrite levels required for the anammox process. According to a recent study, anammox bacteria originated on Earth approximately 2.5 billion years ago, during a major oxygenation event [58]. This places anammox bacteria close to the diversification of nitric reductase into the use of NO and O_2_ as major substrates and the origin of aerobic respiration [58]. Conversion from anaerobic respiration and the dominance of anaerobic processes (including denitrification) to an increasing significance of aerobic functions (including aerobic metabolisms) signified a major milestone in the history of the biogeochemical N-cycle on earth. The stability and effectiveness of processes are maintained by the interactions between anammox bacteria and other microbial communities, such as nitrifies, denitrification enzymes, and heterotrophic bacteria. Ammonia oxidation is supported by nitrifies like Nitrosomonas and Nitrobacter, which produce nitrite, a substrate for anammox bacteria. Anammox activity may be impacted by denitrification enzyme’s competition for nitrite. Furthermore, by altering substrate availability, heterotrophic bacteria can contribute to the breakdown of organic matter, which in turn influences anammox efficiency indirectly [125].

Furthermore, interactions between microbial consortia can improve wastewater treatment systems’ nitrogen removal effectiveness. For example, in a one-stage anammox process, the synergistic interactions between anammox bacteria and partial nitrifies increase the efficiency of nitrogen removal while lowering oxygen demand [160].

## 7. Hydrazine Intermediate Pathways in Anaerobic Oxidations

Anammox bacteria are able to synthesize and break down hydrazine, a highly toxic and energy-rich compound that inhibits other denitrifying microorganisms [145,161]. The bacterial AMO could use hydrazine as an external reductant to provide the electrons needed for activity, enabling the AMO to oxidize a variety of compounds. Its effects reduce nitrate-reducing bacteria (NRB) or nitrite-oxidizing bacteria (NOB), while other bacteria (anammox) can use it as an energy source. Dinitrogen gas is the final product of the HAO’s hydrazine oxidation [162]. The anammox culture is able to convert and tolerate 1 mM hydrazine, with the half-saturation constant and inhibition constant of N_2_H_4_ being 10.42 mg N/L and 1393.88 mg N/L, respectively [163]. Hydrazine is evaluated by anammox activity retrieved by adding a trace amount after exposure, constant continuing-term, to higher concentration of the nitrite [164]. Long-term addition of hydrazine could enhance recovery and expand the autotrophic nitrogen removal capacity in completely autotrophic nitrogen removal over nitrite (CANON) [158,165].

**Figure 7:**
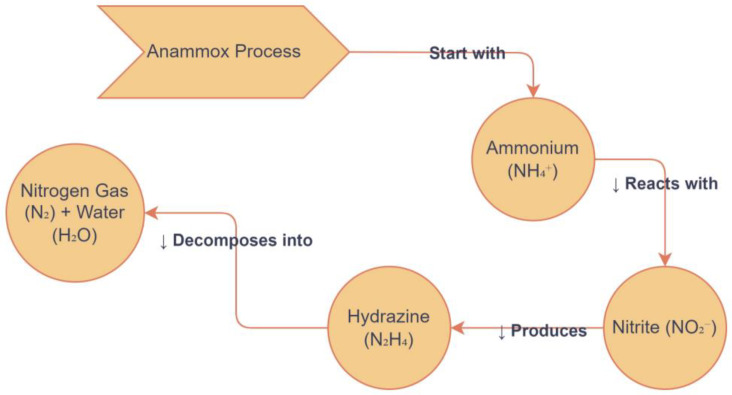
Hydrazine intermediate pathways in anaerobic ammonium oxidation.

Same observations were noted in a previous study with transferring batch experiments and bed biofilm reactors with anammox upgrading values [166]. The electrons freed from exogenous hydrazine oxidation were speculated. Additionally, hydrazine may have exerted an repressive effect on NOB although certainly affecting anammox bacteria, or the hydrazine addition may have increased the nitrite conversion rate in the anammox culture [158]. The pathways of hydrazine intermediate in anaerobic ammonium oxidation are shown in Figure 7. A research study reported that the deterioration rate was improved by both nitrate and ammonium due to exogenous hydrazine in the anammox system, but nitrate accumulation rate was reduced. These findings correspond with those by other researchers: that the anammox moving bed biofilm reactor [165,166] demonstrates the effects of hydrazine addition in CANON processes. However, the addition of hydrazine increases total nitrogen. All these findings vary among processes; in [158], however, 200% increases in CANON were reported; in [165], the increase was 83.3%; in [166], a 20% increase in the moving bed biofilm reactor was detected; in [164], a 30% increase in a pilot-scale sequencing batch reactor was reported. Batch experiments were conducted to study the changes in anammox characteristics under different substrate combinations, allowing for the determination of the exact effects of hydrazine addition on anammox substrate conversion performance. It was found that 5% of the improved SAA was due to increased ammonium removal, 25% to accelerated nitrite degradation, and 12% to reduced nitrate production.

### 7.1. Process Monitoring, Control, and Safety Measures of Hydrazine

The implementation of robust process monitoring control and safety measures is vital for the harmless and efficient operation of hydrazine-mediated anaerobic oxidation systems. Recently, advanced sensor technology has permitted real-time monitoring of hydrazine, nitrite, ammonium, and nitrate concentrations, which provides crucial data for process optimization [132]. Based on this, control systems such as PID controllers have been integrated to maintain optimal substrate ratios, dynamically adjust influent flow rates, aeration levels, and environmental conditions [167]. Moreover, safety engineering plays a vital role with the complete risk mitigation strategies being recommended and implemented [168]. These include the safe installation of vent emissions and gas handling systems as well as pressure relief valves to prevent hazardous conditions and the use of corrosion-resistant materials for long-term structural integrity [57]. Thus, in the long term, these applications further validate these measures. Regarding environmental considerations, the design of closed-loop systems that minimize hydrazine emissions and waste is recommended [132]. Cooperatively, these measures, together with engineering, create a multi-layered framework that guarantees safety, operational efficiency, and environmental responsibility in hydrazine-mediated processes. These unique challenges are posed by hydrazine’s reactivity and toxicity; however, it is possible toharness its potential in wastewater treatment and related applications [138].

### 7.2. Reactor Design and Engineering Optimization

The anammox process relies heavily on efficient reactor design to maximize the contact between ammonium, nitrite, and the specialized bacteria responsible for anaerobic oxidation. Here, we discussed two of the most commonly used reactor types: the moving bed biofilm reactor (MBBR) and the sequencing batch reactor (SBR) which operate in discrete cycles, supporting high biomass retention and efficient nitrogen removal.

MBBR could be an efficient system for the incorporation of biomass as biofilm instead of flocs. A research study compared the pilot MBBR plant against a CAS wastewater plant tested for nitrogen removal efficiency. It was concluded that MBBR reduced plant footprint by 50% compared to a CAS, leading to a capital investment and operational cost reduction that resulted in a lower cost for treated wastewater. Moreover, another study reviewed the slaughterhouse and meat industry wastewater as promising approaches for managing biological treatment technologies. In this research, a laboratory test was conducted to assess the removal efficiencies of COD by 98%, ammonia by 99%, and phosphorus by 65%, while MBBR technology demonstrated a removal efficiency of 81% for nitrogen and 96% for phosphorous [169]. These results are evidence of MBBR technology being more efficient than others. MBBR technology has a high, or very high, rate for nitrogen removal, while MBBR varies from high for synthetic wastewater to medium-high for real wastewater [170]. Moreover, the sequencing batch reactor (SBR) process is the most accepted and common in aerobic treatment technologies. It is used for both municipal wastewater and wastewater treatments for a variety of industries including refineries and petrochemical plants. The difference between SBR and the activated sludge system is that the microbial metabolic breakdown of chemicals in the wastewater influent in SBR is also known as time- rather than space-oriented. In a previously published study, the removal efficiency of nitrate was measured using an SBR plant and am activated sludge plant, with values being 98.1% and 89.7% respectively. The total nitrogen removal efficiency was 84.1% for the SBR plant and 79.7% for the activated sludge plant [134].

In contrast, from an engineering perspective, the MBBR and SBR differ significantly in their design operation and suitability for anammox-based wastewater treatment. MBBR operates as a continuous flow system, utilizing free-floating biofilm carriers to provide a large surface area for microbial growth. The design continuous flow nature of MBBR simplifies process control and reduces sensitivity to fluctuations in influent load, making it a robust and stable system. The modular and scalable design makes it suitable for both large-scale and small-scale applications. Additionally, the SBR operates in discrete batch cycles, encompassing phases such as reacting, filling, decanting, settling, and idling, all within a single reactor. SBR cyclic operation offers greater flexibility in handling varying wastewater loads and precise control of conditions crucial for anammox. The energy consumption in this system tends to be higher due to the need for intermittent aeration and mixing, as well as the energy required for sludge settling and decanting. Scaling up SBR systems can also be challenging, often necessitating multiple reactors to handle large wastewater volumes, which increases the system’s footprint and operational demands [171]. In general, when applied to the anammox process, both systems present different advantages and limitations. The MBBR is preferred for larger-scale anammox applications due to its high biomass retention, which supports the slow growth rate of anammox bacteria [172]. On the other hand, SBRs are more commonly used in laboratory-scale studies, where precise control over operational parameters is required. While SBRs offer flexibility, their complexity and scalability issues make them less favorable for full-scale anammox implementations. In conclusion, from an engineering standpoint, the choice between MBBR and SBR for anammox applications depends on factors such as the scale of operation, desired process stability, energy efficiency, and the level of control required. MBBRs offer a simpler, more resilient, and energy-efficient solution for continuous anammox processes, whereas SBRs provide operational flexibility but require more complex control and higher energy input.

## 8. Advantages and Limitations of Anammox Bacteria for Pharmaceutical Wastewater Treatment

Anammox bacteria could remove N from wastewater with lower energy consumption than conventional nitrification–denitrification processes. The anammox process is different from traditional biological treatment processes; anammox operates under anaerobic conditions, significantly decreasing organic carbon requirements and oxygen demand. It is an important and lower-energy consumption natural process; anammox is a more sustainable option for N removal in wastewater treatment plants [173]. The anammox process generates minimal biomass, reducing sludge handling and disposal costs. In addition, anammox is a low-chemical-input process, which is an eco-friendly and sustainable option for nitrogen removal in wastewater treatment in agro-ecosystems. Moreover, the integration of anammox with denitrifying anaerobic methane oxidation (DAMO) and other microbial processes can enhance nitrogen removal. However, there are certain limitations to consider. Some pharmaceutical compounds, especially antibiotics, can inhibit anammox bacteria, reducing process efficiency. The anammox process also needs specific environmental conditions, such as a pH range of 6.7 to 8.3, temperatures ranging from 25 to 40 °C, and very low levels of dissolved oxygen (DO < 0.5 mg/L) [8]. Even though anammox has been used successfully in the lab, it is still hard to keep its performance stable in full-scale pharmaceutical wastewater treatment because of changes in the input composition.

## 9. Conclusions

The use of anaerobic ammonium oxidation (anammox) processes holds significant promise for the treatment of pharmaceutical wastewater, especially for contaminants such as bisphenol A (BPA) and bisphenol S (BPS). The diversity of anammox bacteria found in natural environments highlights their critical role in the nitrogen cycle, contributing significantly to global nitrogen removal. This study demonstrates the vital role performed by microbial communities, especially anammox bacteria, in decreasing the nitrogen level and facilitating environmental sustainability. This study discusses the pharmaceutical wastewater contamination impact on human health and our ecosystem. Furthermore, we also demonstrate the best treatment and rector wastewater contamination. Moreover, in microbial communities, functional genes hydrazine dehydrogenase and hydrazine synthase play key roles in converting ammonium and nitrite into nitrogen gas. Anammox bacteria’s capacity to adapt to various environmental conditions, particularly their resistance to hazardous chemicals like hydrazine, highlights their promise for sustainable nitrogen management in aquaculture and other wastewater systems. Finally, anammox bacteria can greatly help reduce nitrogen pollution and advance environmentally friendly wastewater treatment techniques. Further research is required to understand the roles of different microbial communities and their functional gene mechanisms in reducing wastewater contamination.

## Figures and Tables

**Table 1:** Removal Efficiency (RE) of 10 Pharmaceuticals and Personal Care Products.

PPCP	RE (%)	Key Studies
Diclofenac	70–99%	High-efficiency removal with GAC and NF [9,101].
Carbamazepine	60–98%	GAC and NF show high efficiency, but variability depends on operating conditions [49].
Ibuprofen	85–99%	Easily removed by GAC and NF due to its hydrophobic nature [102].
Diuron	50–95%	Adsorption pre-treatment significantly improves removal [103].
Gemfibrozil	90–99%	Consistently high-efficiency removal with GAC and NF [9].
Saccharin	70–95%	Moderately efficient removal with NF alone, improved with GAC pre-treatment [103].
Naproxen	90–99%	Highly efficient removal across studies due to its affinity for adsorption [102].
Trimethoprim	80–98%	Effective removal with GAC and NF, but pH-dependent [49].
Triclosan	85–99%	Highly efficient removal due to strong adsorption onto GAC [9].
Benzotriazole	30–99%	Moderately efficient removal with NF alone, improved with GAC pre-treatment [103].

**Table 2:** The nitrogen removal efficiency ranges.

Nitrogen/Removal Types	Experiments	Tempeture/Time	Efficeincy Range	References
Nitrogen	Sequencing Batch Reactor (SBR),	25–35 °C, HRT: 6–24 h	80–90% (up to 95%)	[137]
Ammonium	Moving Bed Biofilm Reactor (MBBR),	30 °C, HRT: 12 h	85–95%	[138]
Total Inorganic Nitrogen	Upflow Anaerobic Sludge Blanket (UASB),	28–32 °C, HRT: 8 h	70–90%	[139]

**Table 3:** Summary of the anammox bacteria discovered to date.

Bacteria	Key Genera	Habitat/Source	References
Brocadia	*Anammoxidans fulgida*	Wastewater treatment plants	[154]
Kuenenia	*stuttgartiensis*	Wastewater treatment systems, natural ecosystems	[155]
Scalindua	*Brodae wagneri sorokinii*	Wastewater/Marine sediments	[147]
Jettenia/other	*asiatica* *Anammoxoglobus propionicus*	Synthetic water	[156]
Anammoximicrobium	*Anammoximicrobium*	Anaerobic reactors	[157]

**Table 4:** The metabolic pathways of anammox functional enzymes.

Enzymes Name	Reaction	Product
HZS	NH_4_^+^ + NO_2_^−^ → N_2_ + 2H_2_O	Dinitrogen gas (N_2_) and water (H₂O)
HDH	N_2_H_4_ → N_2_ + 4H^+^ + 4e^−^	Dinitrogen gas (N_2_) and protons (H^+^)
NIR	NO_2_^−^ + 2H^+^ + e^−^ → NO + H_2_O	Nitric oxide (NO) and water (H_2_O)
HAO	NH_2_OH + H_2_O → NO_2_^−^ + 4H^+^ + 4e^−^	Nitrite (NO_2_^−^), protons (H^+^), and electrons (e^−^)
AMO	NH_3_^+^ + O_2_+2H^+^ + 2^e−^→NH_2_OH + H_2_O	Hydroxylamine (NH_2_OH) and water (H_2_O)

## Data Availability

All relevant data are included in the paper.
